# How Hormonal Balance Changes Lives in Women with Psoriasis

**DOI:** 10.3390/jcm14020582

**Published:** 2025-01-17

**Authors:** Fortunato Cassalia, Anna Lunardon, Giovanni Frattin, Andrea Danese, Francesca Caroppo, Anna Belloni Fortina

**Affiliations:** 1 Dermatology Unit, Department of Medicine (DIMED), University of Padua, 35121 Padua, Italy; anna.lunardon@studenti.unipd.it (A.L.); giovanni.frattin@studenti.unipd.it (G.F.); francesca.caroppo@unipd.it (F.C.); anna.bellonifortina@unipd.it (A.B.F.); 2 Section of Dermatology and Venereology, Department of Medicine, University of Verona, 37126 Verona, Italy; andrea.danese_02@studenti.univr.it; 3 Pediatric Dermatology Regional Center, Department of Women’s and Children’s Health (SDB), University of Padua, 35122 Padua, Italy

**Keywords:** psoriasis, hormonal balance, estrogen, menstrual cycle, pregnancy, menopause, women’s health, quality of life

## Abstract

Psoriasis is a chronic, immune-mediated skin disease significantly impacting women, with disease severity often modulated by hormonal fluctuations. This review examines the influence of hormonal changes on the course of psoriasis in women, focusing on key life stages—including the menstrual cycle, pregnancy, postpartum, and menopause—and their impact on disease progression and symptomatology. Estrogen, the principal female sex hormone, plays a critical role in immune modulation. Variations in estrogen levels, which occur naturally throughout a woman’s life, are associated with fluctuations in psoriasis severity. Low estrogen levels, as seen during menstruation or menopause, are linked to symptom exacerbation, while elevated levels during pregnancy may reduce symptoms in some women. However, responses are variable, with others experiencing no change or worsening during pregnancy. Postpartum, the rapid decline in estrogen often triggers severe flare-ups, while menopause, marked by a sustained estrogen reduction, frequently correlates with increased disease severity and flare frequency. The review also addresses the profound impact of psoriasis on women’s quality of life, including physical discomfort, psychological distress, and social stigma. Additionally, fertility concerns are discussed, as severe psoriasis and associated treatments may increase the risk of adverse pregnancy outcomes. Consideration is given to hormonal therapies, lifestyle modifications, and their effects on psoriasis, underscoring the need for personalized treatment approaches that account for hormonal influences. Understanding these hormonal dynamics is essential for developing targeted, effective management strategies that enhance quality of life for women affected by psoriasis.

## 1. Introduction

Psoriasis is a chronic, immune-mediated dermatologic condition characterized by hyperproliferation of keratinocytes, leading to erythematous, scaly plaques on the skin’s surface [1,2]. Affecting an estimated 125 million individuals globally, psoriasis is far more than a cosmetic issue; it imposes a substantial burden on physical health, mental well-being, and quality of life [3,4]. While both genders are susceptible to psoriasis, recent research highlights the profound influence of hormonal balance on disease manifestation and progression in women, adding complexity to its clinical management [5,6]. Hormonal fluctuations throughout the menstrual cycle, pregnancy, postpartum period, and menopause play a significant role in modulating immune responses and impacting psoriasis severity in women [7,8]. Estrogen and progesterone, among other hormones, interact with immune cells to influence cytokine production, altering inflammatory pathways central to psoriasis pathogenesis [6,9]. High estrogen levels, for example, are typically associated with anti-inflammatory effects, which may alleviate symptoms, while reduced estrogen or elevated prolactin during certain life stages can exacerbate disease activity [5,6]. These hormonal dynamics not only influence the immune milieu but also impact various aspects of a woman’s life, including her emotional health, reproductive decisions, and social relationships [5,6,7,8,9]. This paper investigates the nuanced relationship between hormonal changes and psoriasis in women, examining how estrogen, progesterone, and other hormones modulate immune function, affect disease severity, and alter life quality. By exploring these mechanisms, it offers a basis for personalized therapeutic strategies that address the unique hormonal context of female patients. Tailored management approaches can significantly improve treatment outcomes and quality of life for women with psoriasis, providing a holistic framework that encompasses hormonal influences, psychological support, and patient-centered care. Enhanced understanding of these interactions not only supports clinical advancements but also empowers women with strategies to navigate the complexities of living with chronic autoimmune disease.

## 2. The Role of Hormones in Psoriasis

Psoriasis is a chronic, immune-mediated disorder characterized by dysregulation of immune responses, leading to persistent inflammation and hyperproliferation of keratinocytes [1,2]. Hormonal factors, particularly estrogen and progesterone, exert considerable influence on immune modulation and can significantly affect the course of psoriasis in women [5,6]. This review explores the mechanisms through which these hormones impact immune function and psoriasis severity (Table 1 and Table 2).

Estrogen and immune modulation: estrogen, the primary female sex hormone, not only regulates the reproductive system and secondary sexual characteristics but also plays a critical role in modulating both innate and adaptive immunity [10]. The immunomodulatory properties of estrogen are multifaceted, involving various immune cells and signaling pathways that influence inflammation levels in psoriasis [6,10].

Anti-inflammatory effects of estrogen: elevated estrogen levels are generally associated with anti-inflammatory effects [6]. Estrogen interacts with estrogen receptors (ERα and ERβ) on multiple immune cells, including T cells, B cells, dendritic cells, and macrophages [11]. Through these receptors, estrogen can downregulate the production of pro-inflammatory cytokines, such as tumor necrosis factor-alpha (TNF-α), interleukin-6 (IL-6), and interferon-gamma (IFN-γ), all of which are central to the pathogenesis of psoriasis. By inhibiting these cytokines, estrogen reduces inflammatory responses and may ameliorate psoriasis symptoms [5,6,11,12].

Additionally, estrogen promotes the secretion of anti-inflammatory cytokines, including interleukin-10 (IL-10) and transforming growth factor-beta (TGF-β). These cytokines modulate immune responses by restricting the activity of pro-inflammatory cells and promoting immune tolerance, which is essential in mitigating autoimmune responses associated with psoriasis [6,13,14,15].

Influence on immune cell function: estrogen also plays a role in altering the balance of T helper (Th) cell subsets. It can shift the immune response from a Th1 and Th17 profile, which is pro-inflammatory and prevalent in psoriasis, to a more anti-inflammatory Th2 profile [16]. This shift reduces the production of inflammatory mediators, subsequently decreasing skin lesions [6,16]. Furthermore, estrogen enhances the activity of regulatory T cells (Tregs), which are pivotal in maintaining immune tolerance and preventing autoimmunity. By enhancing Treg function, estrogen contributes to the suppression of autoimmune processes involved in psoriasis pathogenesis [15,16,17].

Progesterone: progesterone, another critical female hormone, plays a vital role in immune regulation by fostering an anti-inflammatory environment, particularly within the context of autoimmune diseases like psoriasis. It achieves this by inhibiting the proliferation of T cells—immune cells that are heavily involved in the inflammatory cascade of psoriasis [9]. T cells, especially T helper (Th1 and Th17) cells, are known for driving pro-inflammatory responses in psoriasis by releasing cytokines such as IL-17, IL-22, and TNF-α, which stimulate keratinocyte hyperproliferation and sustain the characteristic plaques and scaling of psoriatic skin [18]. Progesterone dampens this activity by reducing the production of these pro-inflammatory cytokines, thereby directly countering immune responses that exacerbate psoriasis [9].

In addition to its impact on T cell function, progesterone modulates immune responses through its influence on other cytokines and immune cells. For instance, it enhances the production of anti-inflammatory cytokines like IL-10, which helps to limit the activity of pro-inflammatory cells, promoting a more regulated immune environment [15,19]. By reducing the expression of inflammatory mediators, progesterone helps restore balance in the immune system, creating conditions that are less favorable for psoriatic inflammation [9,15,19]. The immunosuppressive effects of progesterone may also explain the symptomatic changes often observed in women with psoriasis during different stages of the menstrual cycle and pregnancy [19]. During the luteal phase of the menstrual cycle, progesterone levels rise, which may contribute to a reduction in psoriatic inflammation and an improvement in symptoms for some women [19,20,21]. In pregnancy, progesterone levels increase significantly, especially in the second and third trimesters, creating an anti-inflammatory state that can lead to symptomatic relief for many women with psoriasis [7]. However, in the postpartum period, when progesterone levels drop sharply, a rebound increase in inflammation may occur, often leading to psoriasis flare-ups [19,20,21]. Understanding these cyclical changes in progesterone and their effects on immune regulation is crucial in managing psoriasis in women, particularly those with hormone-sensitive disease patterns. This insight suggests that hormonal fluctuations are a significant factor in disease severity and may offer a basis for exploring hormone-based therapeutic approaches to modulate immune responses and improve patient outcomes in women with psoriasis.

Prolactin: prolactin, a polypeptide hormone primarily associated with lactation, is increasingly recognized for its role in immune regulation. Prolactin is produced not only by the pituitary gland but also by various immune cells, including T and B lymphocytes, under certain conditions [22]. This hormone has pro-inflammatory properties that may exacerbate autoimmune conditions like psoriasis [22,23].

In psoriasis, elevated prolactin levels have been linked to an increase in keratinocyte proliferation—a hallmark feature of the disease [23]. Prolactin promotes the expression of pro-inflammatory cytokines, such as TNF-α, IL-6, and IL-17, in keratinocytes and immune cells. These cytokines drive inflammatory processes in the skin, leading to the formation and maintenance of psoriatic plaques. Prolactin can also stimulate lymphocyte activity, enhancing the immune response and potentially worsening psoriasis symptoms [22,23,24]. Understanding prolactin’s role in psoriasis underscores the need for therapies that modulate this hormone in patients who show elevated levels, especially during times of physiological stress or lactation.

Cortisol and stress response: cortisol is a glucocorticoid hormone synthesized by the adrenal glands in response to stress through the hypothalamic–pituitary–adrenal (HPA) axis. It plays a complex role in immune regulation and inflammation. Acute increases in cortisol typically exert immunosuppressive effects by inhibiting the release of pro-inflammatory cytokines and reducing lymphocyte proliferation, which are beneficial for transient inflammatory responses. However, chronic stress leads to dysregulation of cortisol production, often resulting in a “flattened” diurnal rhythm where cortisol levels are abnormally low in the morning and elevated at night [25,26].

This cortisol dysregulation can shift the immune system into a chronic pro-inflammatory state, exacerbating autoimmune conditions like psoriasis. When cortisol is persistently low or irregular, the body’s natural anti-inflammatory response weakens, allowing inflammatory pathways, particularly those involving IL-17 and TNF-α, to persist unchecked [27,28]. This heightened inflammatory response in the skin leads to increased keratinocyte activity and further contributes to plaque formation and scaling in psoriasis [27,28]. The relationship between cortisol levels, stress, and psoriasis suggests that stress management strategies—such as mindfulness-based interventions, cognitive-behavioral therapy, and, in some cases, pharmacological support—could play a supportive role in managing psoriasis [29].

Hormonal imbalance and psoriasis severity: the delicate balance between hormones such as estrogen, progesterone, prolactin, and cortisol is crucial for immune homeostasis, and disruptions in this balance are known to influence psoriasis severity [5,6,18,22,23,28]. Estrogen generally promotes anti-inflammatory effects, while progesterone supports immune tolerance; together, they create a protective hormonal environment against autoimmune inflammation [6,9]. However, when estrogen levels are low (e.g., during the luteal phase of the menstrual cycle or postmenopause), or when there is an imbalance between estrogen and progesterone, the risk of immune dysregulation increases [6,10]. This imbalance can shift the immune response towards a pro-inflammatory state, marked by an increase in Th1 and Th17 activity, both of which are associated with psoriasis pathogenesis [16]. In clinical practice, understanding these hormonal interdependencies underscores the importance of a personalized approach to psoriasis treatment in women. Comprehensive management strategies that address hormonal balance, stress reduction, and lifestyle modifications hold promise for improving quality of life and reducing disease severity in women affected by psoriasis [30].

Testosterone: testosterone, a key male hormone, appears to play a significant role in modulating psoriasis. Testosterone is known to promote an immune shift toward a Th2 phenotype, which counteracts the Th1/Th17-dominant response observed in psoriasis. Recent studies indicate that testosterone levels are significantly lower in male patients with psoriasis compared with healthy controls, suggesting a potential pathogenic and therapeutic role for this hormone [31]. However, the correlations between testosterone levels and the Psoriasis Area Severity Index (PASI) remain partially understood, requiring further investigation. Cemil et al. did not observe a significant correlation between testosterone levels and PASI scores, suggesting that the relationship between testosterone and psoriasis severity may be mediated by complex factors that are not yet fully understood [31]. Another study by Allam et al., involving 121 male patients with psoriasis, found an association between low testosterone levels and high PASI scores. This suggests that testosterone may have an immunomodulatory role, reducing inflammation and the clinical severity of psoriasis [32]. These findings indicate a complex role for testosterone in psoriasis. Reduced testosterone levels in male patients may contribute to an unbalanced immune activation favoring a Th1/Th17 phenotype, thereby exacerbating cutaneous inflammation. However, the inconsistent correlation between testosterone and psoriasis severity across studies highlights the need for further research to clarify the underlying mechanisms [31,32].

**Table 1:** Role of hormones in immune modulation.

Hormone	Effects	References
Estrogen	- Suppresses production of pro-inflammatory cytokines: tumor necrosis factor-alpha (TNF-α), interleukin-6 (IL-6), and interferon-gamma (IFN-γ). - Promotes the production of anti-inflammatory cytokines: interleukin-10 (IL-10) and transforming growth factor-beta (TGF-β). - Switches the balance from a pro-inflammatory Th1 and Th17 response to a more anti-inflammatory Th2 response. - Enhances Treg function.	[5,6,11,12] [6,13,14,15] [16] [17]
Progesterone	- Dampens CD4+ T cell activation. - Inhibits proliferation of T cells. - Reduces production of pro-inflammatory cytokines (such as TNF and IL-2).	[9] [9]
Prolactin	- Promotes keratinocyte proliferation. - Promotes production of pro-inflammatory cytokines, such as TNF-α, IL-6, and IL-17, in keratinocytes and immune cells. - Stimulates lymphocyte activity.	[23] [22,23,24]
Cortisol	- Acute cortisol release has immunosuppressive effects, inhibiting the release of pro-inflammatory cytokines and reducing lymphocyte proliferation. - Chronic stress can lead to dysregulation of cortisol production and promotes a pro-inflammatory state with an increase in production of pro-inflammatory cytokines (such as IL-17 and TNF-α).	[25,26] [25,26,27,28]
Testosterone	- Promote an immune shift toward a Th2 phenotype, which counteracts the Th1/Th17-dominant response.	[31,32]

**Table 2:** Impact of hormones in psoriasis.

Hormone	Effect on Psoriasis	References
Estrogen	Elevated estrogen levels are associated with anti-inflammatory effects, and therefore lead to an improvement of psoriasis.	[5,6,11,12,13,14,15]
Progesterone	Progesterone has an immunosuppressive effect, with an improvement in psoriasis symptoms.	[9]
Prolactin	Elevated prolactin levels have been linked to an increase in keratinocyte proliferation and an increase in the expression of pro-inflammatory cytokines, and therefore lead to a worsening of psoriasis.	[22,23,24]
Cortisol	Acute increases in cortisol have immunosuppressive effects and lead to an improvement in psoriasis symptoms. The dysregulation of cortisol production (due to chronic stress), often results in cortisol levels abnormally low in the morning and elevated at night, and this dysregulation can shift the immune system into a chronic pro-inflammatory state, exacerbating psoriasis.	[25,26,27,28]
Testosterone	Reduced testosterone levels in male patients may contribute to an unbalanced immune activation favoring a Th1/Th17 phenotype, thereby exacerbating cutaneous inflammation. There is an inconsistent correlation between testosterone and psoriasis severity across studies	[31,32]

## 3. Hormonal Fluctuations Across Life Stages

Hormonal fluctuations throughout a woman’s life can influence the severity and progression of psoriasis due to their immunomodulatory effects. Key hormonal events—including the menstrual cycle, pregnancy, the postpartum period, and menopause—induce shifts in estrogen and progesterone levels, which can impact psoriasis symptoms differently [7]. Understanding these influences is essential for tailoring treatment approaches to optimize care at each life stage (Table 3).

### 3.1. Menstrual Cycle

The menstrual cycle is a monthly series of hormonal changes, particularly involving estrogen and progesterone, which influence immune responses and inflammation. Many women with psoriasis report symptom variation during different phases of their cycle [33].

Symptom worsening during luteal phase and menstruation: in the luteal phase (post-ovulation) and menstruation, estrogen and progesterone levels decline significantly, reducing their anti-inflammatory effects. This hormonal drop is linked to increased inflammation, often exacerbating psoriasis symptoms such as itching, redness, and scaling [33]. Research suggests that low estrogen levels may increase pro-inflammatory cytokine activity, such as TNF-α and IL-6, which are key players in psoriasis pathogenesis [11,12].

Clinical implications: recognizing this cyclical pattern enables healthcare providers to implement proactive strategies, such as temporary increases in topical treatments or lifestyle adjustments, to mitigate flare-ups during high-risk phases of the menstrual cycle. Lifestyle modifications and stress management may also enhance symptom control and quality of life [7,19,34].

**Table 3:** Hormonal fluctuation across women’s life stages.

Women’s Life Stages	Effect on Estrogen and Progesterone Levels	Effect on Psoriasis Symptoms	Ref
Menstrual cycle (luteal phase and menstruation)	Decline in estrogen and progesterone levels.	Increased inflammation and exacerbation of psoriasis symptoms.	[31]
Pregnancy	Elevated levels of estrogen and progesterone.	Improvement in psoriasis symptoms. Sometimes complete remission.	[6,8,9,34]
Postpartum period	Rapid decline in estrogen and progesterone levels.	Rebound effect with flare-ups and worsening of psoriasis, sometimes more severe than before pregnancy.	[7,8,9,19]
Menopause	Decline in estrogen and progesterone production.	Increased psoriasis activity with more frequent and severe flare-ups during and after menopause.	[7,8,10]

### 3.2. Pregnancy

Pregnancy induces substantial hormonal shifts, with estrogen and progesterone levels rising progressively to maintain the pregnancy and promote fetal development. These elevated hormone levels can profoundly impact immune function and psoriasis symptoms [6,9,19].

Improvement in symptoms: during pregnancy, many women with psoriasis experience symptom relief due to the anti-inflammatory effects of high estrogen and progesterone [6,8,9]. Estrogen has been shown to modulate immune responses by promoting an anti-inflammatory environment, decreasing levels of pro-inflammatory cytokines like TNF-α and IL-6, and fostering regulatory T cell activity [11,12]. Research indicates that up to 60% of pregnant women with psoriasis may see an improvement or even achieve remission [34].

Variability in response: despite the general trend of symptom improvement, a subset of women may experience no change or worsening symptoms. This variability could be due to genetic factors, environmental influences, and individual differences in immune system sensitivity [34].

Immune system shift: pregnancy involves a shift toward a Th2 immune response, which is less inflammatory and beneficial for psoriasis. This shift decreases Th1 and Th17 responses—typically dominant in psoriasis—and helps prevent excessive inflammation, contributing to symptom improvement [35].

Clinical management: managing psoriasis in pregnant women requires a careful balance between effective symptom control and fetal safety. Topical therapies are generally preferred, while systemic treatments are used cautiously due to potential risks. Collaboration between dermatologists and obstetricians is vital for optimal care, as psoriasis treatment must be tailored to minimize risks to the mother and baby [9,31,34].

### 3.3. Postpartum Period

The postpartum period involves a sudden decline in estrogen and progesterone levels as the body returns to its pre-pregnancy state. This sharp hormonal drop can trigger a “rebound effect”, which may lead to significant symptom flare-ups.

Rebound effect and symptom flare-ups: this hormonal shift is associated with increased production of pro-inflammatory cytokines, leading to heightened immune activity. Many women experience more severe psoriasis symptoms post-delivery, sometimes presenting with new lesions or more extensive skin involvement. This period is challenging as women balance new motherhood responsibilities while managing their health [7,8,9,19].

Contributing factors: in addition to hormonal fluctuations, factors like sleep deprivation, physical recovery from childbirth, and postpartum stress can exacerbate psoriasis. Psychological aspects, including postpartum depression and anxiety, may also worsen disease severity, emphasizing the need for mental health support [36].

Treatment considerations: postpartum psoriasis management should take a holistic approach, focusing on physical and emotional health, breastfeeding considerations, and social support. Topical therapies are generally preferred for their safety profile in breastfeeding mothers. If systemic treatments are required, healthcare providers must carefully weigh the benefits against potential risks for both mother and infant [36].

### 3.4. Menopause

Menopause signifies the end of reproductive years and brings a permanent reduction in estrogen and progesterone levels. These hormonal declines can have significant implications for psoriasis severity and symptom management [7,8,10].

Increased disease activity: with the loss of estrogen’s anti-inflammatory properties, many women experience more frequent or severe psoriasis flare-ups during menopause. Estrogen deficiency is linked to increased production of pro-inflammatory cytokines and decreased regulatory T cell function, creating an immune environment conducive to psoriasis exacerbation. Lesions may become more extensive or resistant to previously effective treatments [7,8,10].

Skin changes and sensitivity: menopause also induces changes in skin texture, such as dryness, thinning, and reduced elasticity. These alterations can worsen psoriasis symptoms by making the skin more susceptible to irritation, discomfort, and heightened sensitivity to treatments. Reduced skin barrier function in menopausal women with psoriasis may lead to an increased risk of injury or infection [7].

Hormone replacement therapy (HRT): hormone replacement therapy (HRT) may offer symptom relief by restoring estrogen levels, potentially mitigating psoriasis severity. Some studies indicate that HRT may alleviate menopausal symptoms and improve psoriasis outcomes. However, the effects of HRT on psoriasis vary among individuals, and HRT carries potential risks, including an increased risk of cardiovascular disease and breast cancer. A comprehensive evaluation of HRT’s benefits and risks is essential in managing menopausal women with psoriasis [36].

#### Clinical Recommendations

In postmenopausal women with psoriasis, healthcare providers should consider revising treatment strategies to account for hormonal changes and skin sensitivities. Adjustments may involve optimizing topical treatments, emphasizing skincare to address dryness and sensitivity, and evaluating the potential role of HRT in symptom management [7,34,37].

## 4. Impact on Quality of Life

Psoriasis significantly affects women’s quality of life by influencing physical health, emotional well-being, social interactions, and professional activities. Hormonal fluctuations often intensify these challenges, particularly during life stages such as menstruation, pregnancy, postpartum, and menopause, which can exacerbate symptoms [30,38].

Physical effects: psoriasis is associated with several physical symptoms that can impair day-to-day functioning.

Discomfort and pain: women with psoriasis frequently experience persistent itching, pain, and skin tightness, which can worsen during hormonal fluctuations. Lesions in sensitive areas such as the scalp, nails, genitals, and skin folds can interfere with routine activities like dressing, grooming, and hygiene. Discomfort can disrupt sleep, leading to fatigue, which further diminishes quality of life. Sleep deprivation can exacerbate both physical symptoms and psychological distress, creating a cycle that compounds the disease burden [39].

Functional impairment: psoriasis can affect fine motor skills, particularly if lesions are present on the hands or if psoriatic arthritis is involved. Nail psoriasis, for instance, may impair tasks such as typing, writing, or buttoning clothes. Joint pain and stiffness, characteristic of psoriatic arthritis, can limit mobility and make simple movements painful. This functional impairment impacts independence and productivity in both personal and professional contexts [40,41].

### Psychological and Emotional Well-Being

Psoriasis has profound effects on mental health and self-perception.

Self-esteem and body image: the visibility of psoriatic lesions can cause psychological distress and impact body image. Women with psoriasis may feel embarrassed, self-conscious, and stigmatized, affecting their confidence in social, professional, and intimate settings. This can result in social withdrawal and reluctance to engage in activities that reveal affected skin, such as swimming or exercising in public spaces [42].

Mental health disorders: women with psoriasis are at an increased risk of mental health conditions, particularly depression and anxiety. The chronic nature of psoriasis, combined with hormonal imbalances and the unpredictability of symptom flare-ups, can exacerbate these mental health challenges. Depression and anxiety are not only consequences of physical symptoms but also contribute to a cycle that can worsen psoriasis severity due to stress and emotional strain [43].

Coping mechanisms: some women may develop maladaptive coping mechanisms in response to psoriasis, such as social withdrawal, substance use, or disordered eating. These behaviors can provide temporary relief from emotional pain but ultimately worsen physical health and mental well-being. Access to mental health support and counseling is crucial for promoting adaptive coping strategies and breaking this cycle [44].

Social and interpersonal relationships: the social implications of psoriasis extend beyond self-perception, affecting relationships and interactions with others:

Social isolation due to the visibility of lesions and the fear of judgment or misunderstanding, many women with psoriasis withdraw from social activities. Misconceptions about psoriasis being contagious can amplify feelings of isolation, as friends, colleagues, or even strangers may avoid physical contact. This isolation can deepen psychological distress and reinforce the negative self-image [45].

Intimate relationships: psoriasis can affect intimate relationships on both physical and emotional levels. Concerns about appearance, physical discomfort during sexual activity, and decreased libido (influenced by hormonal changes and psychological distress) can impact intimacy. Open communication with partners is essential for managing expectations and providing reassurance. Educating partners about psoriasis’s non-contagious nature can also foster understanding and empathy, strengthening relationships [45,46,47].

Communication and education: educating close contacts about psoriasis and its impact can dispel misconceptions and improve social support. Friends and family who understand the chronic, non-contagious nature of psoriasis are better equipped to provide meaningful support, reducing the emotional burden and promoting acceptance [48].

Professional and educational impact: psoriasis also affects women’s professional lives and educational pursuits, presenting barriers to productivity, acceptance, and advancement.

Work productivity: physical symptoms such as pain and fatigue, combined with psychological distress, can reduce productivity, increase absenteeism, and limit career opportunities. Psoriatic arthritis may further hinder workplace performance by restricting mobility and causing joint pain, especially in physically demanding jobs. Visible lesions can be particularly challenging in professions that emphasize appearance or require public interaction, as stigma and discomfort can affect performance and self-confidence [45,49,50].

Discrimination and stigma: women with psoriasis may encounter discrimination or harassment in the workplace or educational settings due to misconceptions about their condition. Psoriasis is often misunderstood, with some perceiving it as contagious or reflective of poor hygiene, leading to prejudice. Legal protections may be necessary to address workplace discrimination, ensure fair treatment, and promote an inclusive environment [51,52].

Academic challenges: managing psoriasis symptoms and treatment regimens can interfere with academic responsibilities, impacting performance and opportunities for advancement. For students with psoriasis, navigating treatment schedules alongside coursework and social obligations may be challenging. Educators and academic institutions that are informed about psoriasis can help provide support and accommodations, such as flexibility with assignments or exams, allowing students to manage their health without compromising their education [53,54].

## 5. Fertility and Reproductive Health

Although psoriasis itself does not directly lead to infertility, several factors associated with the condition can influence reproductive health, including the impact of systemic inflammation, medications, stress, and lifestyle factors. Here is how each of these elements may affect fertility.

Disease severity and systemic inflammation: psoriasis is an inflammatory disease that, in severe cases, can lead to systemic inflammation beyond the skin. This widespread inflammation can disrupt reproductive function by influencing hormonal regulation, ovulation, and even implantation [55,56].

Hormonal disruption: chronic inflammation can interfere with the hypothalamic–pituitary–ovarian (HPO) axis, which is crucial for regulating reproductive hormones like estrogen, progesterone, and luteinizing hormone (LH). Disruptions in this axis can lead to irregular menstrual cycles, affecting fertility by making it harder for ovulation to occur predictably [6,8,9,25,55,56].

Impact on ovulation and implantation: inflammation may also affect the uterus and ovaries directly, impairing ovulation or creating a less hospitable environment for implantation. Studies have shown that elevated inflammatory markers (e.g., TNF-alpha, IL-6) can influence the reproductive environment, potentially lowering conception rates or increasing the risk of early miscarriage in women with chronic inflammatory conditions [12,54,57,58].

### 5.1. Medication Effects

Some medications used to treat moderate-to-severe psoriasis have known teratogenic effects, meaning they can cause birth defects and are generally unsafe during pregnancy. This is particularly true for medications such as methotrexate and retinoids [59].

Methotrexate: methotrexate is a systemic immunosuppressive drug that is often used to treat severe psoriasis but is highly teratogenic. It interferes with folate metabolism, which is crucial for DNA synthesis and cell division, affecting both male and female fertility. Women planning a pregnancy are advised to discontinue methotrexate for at least 3 months prior to conception [59].

Retinoids (e.g., acitretin): retinoids are derivatives of vitamin A that are used in psoriasis management but can cause severe birth defects if used during pregnancy. Women on retinoids are usually advised to wait at least 3 years after stopping treatment before attempting to conceive due to the drug’s long half-life [59].

Other immunosuppressants: some other medications, like cyclosporine, may be used in specific cases but require close monitoring due to potential risks to the developing fetus and possible impacts on fertility [59]. Biologic drugs such as TNF inhibitors are generally considered safer for use during pregnancy, but each case should be evaluated individually [59].

### 5.2. Post-Vaccination Psoriasis Flare Differences Between Male and Female Patients

Karampinis et al. found that there were more cases of psoriatic male patients with long post-vaccination periods of flare than psoriatic women (period over 30 days), as well as more cases of female patients whose post-vaccination period flare occurrence did not exceed 7 days (F: 19, M: 11), meaning that female patients tend to have flare-ups more quickly after the trigger. This observation is likely driven by hormonal influences on the immune system and tissue repair mechanisms. Estrogen appears to confer more efficient anti-inflammatory and wound-healing properties, while testosterone may contribute to prolonged inflammation and slower recovery [60].

### 5.3. Psychological Stress and Hormonal Balance

The stress of living with a chronic condition like psoriasis can also play a role in reproductive health.

Stress and the HPO axis: psychological stress can lead to changes in the HPO axis, impacting hormone release and potentially leading to menstrual irregularities, anovulation (absence of ovulation), or luteal phase defects (which can hinder implantation). Chronic stress activates the hypothalamic–pituitary–adrenal (HPA) axis, increasing cortisol levels. Elevated cortisol can disrupt the delicate balance of reproductive hormones, further complicating fertility [25,55,56].

Cyclical impact of flare-ups: psoriasis symptoms often fluctuate with hormonal changes, and flare-ups may exacerbate stress levels, creating a cyclical problem. The additional stress during flare-ups may impact reproductive hormones, increasing the likelihood of irregular periods and complicating timing for conception [31,55].

### 5.4. Lifestyle Factors

Psoriasis is associated with certain lifestyle factors, including higher rates of obesity, smoking, and alcohol consumption, which can adversely impact reproductive health.

Obesity and insulin resistance: obesity is prevalent among individuals with psoriasis and is a known risk factor for insulin resistance, which can contribute to polycystic ovary syndrome (PCOS). PCOS is a common endocrine disorder that can cause irregular menstrual cycles and anovulation, both of which can negatively impact fertility [59,61].

Smoking and alcohol: smoking and excessive alcohol use are also more common in individuals with psoriasis and have both been linked to decreased fertility. Smoking can lead to premature ovarian failure, reduced egg quality, and increased risk of early pregnancy loss. Alcohol, on the other hand, can interfere with hormone levels and reduce the likelihood of conception [60,61,62,63].

Metabolic syndrome: psoriasis is also linked to metabolic syndrome, which is a combination of conditions including hypertension, hyperglycemia, and abnormal cholesterol levels. Metabolic syndrome can contribute to inflammation and hormonal dysregulation, further complicating fertility and reproductive health [59,64].

### 5.5. Pregnancy Planning and Management

For women with psoriasis, careful planning is essential for a healthy pregnancy. This includes consulting healthcare providers about safe medication use, managing disease activity, and addressing any lifestyle modifications that could improve fertility outcomes.

Preconception counseling: women with psoriasis should consult with healthcare providers before trying to conceive to discuss medication adjustments and other preparation steps. Discontinuing certain medications, switching to safer alternatives, or modifying the dosage under medical supervision may be necessary to ensure a safe pregnancy [59,65].

Management during pregnancy: in some cases, pregnancy can improve psoriasis symptoms due to hormonal changes that favor an anti-inflammatory state. However, managing psoriasis during pregnancy can be challenging because options for systemic treatments are limited. Topical treatments are generally safer and are often preferred for controlling symptoms [7,59,65].

Lifestyle adjustments: for women planning pregnancy, addressing lifestyle factors such as weight management, smoking cessation, and alcohol reduction is critical. Optimizing these aspects can improve fertility, reduce inflammation, and enhance overall reproductive outcomes [59,62,63].

## 6. Management Strategies

Effective psoriasis management in women requires a personalized treatment approach that considers unique factors, such as hormonal fluctuations, disease severity, comorbidities, and life stages. Here is a breakdown of scientifically supported approaches in managing psoriasis.

### 6.1. Topical Therapies

Corticosteroids: topical corticosteroids are often the first-line treatment for mild-to-moderate psoriasis. They help reduce inflammation and slow cell turnover. However, long-term use should be monitored to prevent skin thinning, especially in sensitive areas [8,66].

Calcineurin inhibitors (e.g., tacrolimus): effective for delicate skin areas like the face and genitals, these agents modulate the immune response without the same side effects as steroids, although they are usually used as second-line treatments [59].

Emollients and moisturizers: regular use of emollients helps keep the skin hydrated, reducing scaling and discomfort. Studies show that well-hydrated skin can improve the efficacy of other topical treatments [67].

### 6.2. Systemic Treatments

Methotrexate and cyclosporine: these immunosuppressants are often used for moderate-to-severe cases of psoriasis but can have significant side effects, including liver and kidney toxicity. For women planning pregnancy, methotrexate is contraindicated due to its teratogenic effects [59].

Acitretin: a retinoid used in psoriasis treatment, acitretin is effective but also teratogenic, with pregnancy avoidance recommended for 3 years post-treatment [59].

### 6.3. Biologic Therapies and Pregnancy

The use of biologic therapies for women with psoriasis who are planning pregnancy or are pregnant is a rapidly evolving area of research and clinical practice [68]. Biologics have revolutionized the management of moderate-to-severe psoriasis by specifically targeting immune pathways involving tumor necrosis factor-alpha (TNF-α), interleukin-17 (IL-17), and interleukin-23 (IL-23) [59]. However, their use during pregnancy requires careful consideration to balance managing maternal disease activity while ensuring the safety of both the mother and the developing fetus. For women with severe psoriasis, disease control during pregnancy is essential to prevent complications such as physical discomfort, psychological stress, and an increased risk of adverse pregnancy outcomes, including preterm delivery and low birth weight [68]. Biologic therapies provide a targeted approach with fewer systemic side effects compared with traditional therapies, such as methotrexate, which is contraindicated during pregnancy due to its teratogenic effects [59,68]. Among biologics, TNF-α inhibitors, such as etanercept, adalimumab, and infliximab, have the most established safety profiles during pregnancy. These medications are classified as FDA pregnancy category B, meaning there is no evidence of risk in animal studies, but human data are limited. They have been successfully used in pregnant women with other autoimmune conditions, such as rheumatoid arthritis and inflammatory bowel disease [69]. The molecular structure of biologics also influences their safety during pregnancy. Large monoclonal antibodies, such as TNF-α inhibitors, are transported across the placenta starting in the second trimester via the neonatal Fc receptor. As a result, fetal exposure is minimal during the first trimester but increases later in pregnancy, raising concerns about potential immunosuppression in the neonate, particularly if biologics are continued into the third trimester. To minimize fetal exposure while maintaining maternal disease control, current guidelines typically recommend discontinuing biologics after the second trimester. However, recent data suggest that biologics with shorter half-lives or lower placental transfer, such as certain TNF-α inhibitors, may be safer for continued use [69]. The safety profiles of newer biologics targeting IL-17 (e.g., secukinumab, ixekizumab) and IL-23 (e.g., guselkumab, risankizumab) during pregnancy are less well established due to limited human data. While preliminary animal studies have shown no teratogenic effects, human studies are lacking [68,70]. Until more robust evidence becomes available, these agents are generally avoided during pregnancy, with TNF-α inhibitors being the preferred biologic choice for women requiring treatment [69]. Biologic therapies are also an important consideration for women planning to breastfeed. Since large protein molecules, such as biologics, are minimally excreted into breast milk and are unlikely to be absorbed by the infant’s gastrointestinal tract due to their size and degradation by digestive enzymes, available evidence suggests that TNF-α inhibitors are compatible with breastfeeding [69,70]. However, decisions regarding breastfeeding should be individualized and involve shared decision-making between the patient and healthcare provider. Preconception counseling is vital for women with psoriasis who are considering biologic therapy. Counseling should address the timing of pregnancy relative to the biologic treatment cycle, the potential need for switching therapies, and the management of psoriasis flares during pregnancy [71]. For women with severe disease who discontinue biologics during the third trimester, alternative therapies, such as phototherapy, can be used to maintain disease control [71,72]. Multidisciplinary care involving dermatologists, obstetricians, and maternal–fetal medicine specialists is essential to optimize outcomes. Despite significant advancements, there are still gaps in understanding the use of biologics during pregnancy. Longitudinal studies tracking pregnancy and neonatal outcomes in women exposed to newer biologics are necessary to establish their safety and efficacy profiles. The development of registry-based studies and post-marketing surveillance programs can provide valuable real-world data on pregnancy outcomes. Additionally, identifying biomarkers that predict maternal and fetal responses to biologics may enhance personalized treatment approaches [69,71]. The use of biologic therapies in women planning pregnancy or who are pregnant requires a nuanced, evidence-based approach. While TNF-α inhibitors remain the safest biologic option during pregnancy, emerging data on IL-17 and IL-23 inhibitors show promise in expanding therapeutic choices [59,69,70]. Effective disease management not only improves maternal quality of life but also contributes to better pregnancy outcomes [71]. Collaboration between healthcare providers and patients is crucial to developing individualized treatment plans that address both maternal and fetal needs, ensuring optimal care throughout the reproductive journey.

### 6.4. Phototherapy

Narrowband UVB and PUVA therapy: phototherapy is effective for moderate-to-severe psoriasis and is considered safe during pregnancy, making it a valuable option for women needing more than topical treatments. Narrowband UVB therapy has been shown to reduce flare-ups without systemic side effects, making it suitable for long-term management [72].

### 6.5. Hormone-Based Therapies

Hormone replacement therapy (HRT): postmenopausal women may see some improvement in psoriasis symptoms with HRT, as estrogen can have anti-inflammatory effects. However, HRT carries risks (e.g., cardiovascular disease, breast cancer), and its impact on psoriasis varies; thorough risk assessment is essential before initiation [36].

### 6.6. Lifestyle Modifications

Stress management/mindfulness, relaxation and meditation: these techniques help manage psychological stress, which can trigger or exacerbate psoriasis flares. Stress management has been shown to positively impact immune function and hormonal balance, potentially reducing symptom severity [69,70].

Cognitive-behavioral therapy (CBT): CBT helps women develop effective coping mechanisms and reduces psoriasis-related anxiety and depression. Studies have found CBT beneficial in reducing stress-related flare-ups [69,70].

Healthy diet/anti-inflammatory diet: diets rich in antioxidants, omega-3 fatty acids, and anti-inflammatory foods, such as fruits, vegetables and whole grains, have been associated with reduced psoriasis symptoms. Omega-3 fatty acids, found in fish and flaxseeds, have been shown to reduce systemic inflammation [71,72].

Avoiding processed foods and alcohol: alcohol, processed foods, and high sugar intake are linked to increased inflammation and can exacerbate psoriasis. A healthy diet supports overall immune function and may reduce the frequency of flares [61,63,73].

Physical activity: regular exercise improves cardiovascular health, reduces obesity risk, lowers stress levels, and improves psoriasis severity. Exercise also reduces pro-inflammatory markers, aiding in better disease management [74].

Personal triggers: common triggers include smoking, alcohol, allergens, and certain foods. Keeping a symptom diary to identify triggers can help in managing flare-ups more effectively. Smoking, for instance, is associated with higher psoriasis risk and severity, making cessation highly beneficial [61,63,75,76,77,78,79].

### 6.7. Multidisciplinary Care

Collaborative approach/team-based care: a multidisciplinary approach involving dermatologists, rheumatologists, gynecologists, mental health professionals, and primary care providers offers comprehensive care. This collaborative care model is critical for women with psoriasis, addressing physical symptoms, emotional challenges, hormonal influences, and reproductive health needs [65,80,81,82].

Patient education/self-management education: educating women about how hormonal changes impact psoriasis empowers them to manage their condition proactively. Support groups, counseling, and access to educational resources can improve self-management and treatment adherence, particularly during life stages associated with hormonal changes, such as pregnancy or menopause [48,65].

Psychological support/mental health access: psychological support is crucial for managing psoriasis, as the visible nature of the disease can lead to anxiety, depression, and social withdrawal. Access to mental health resources like counseling and support groups can help women manage the emotional aspects of living with a chronic skin condition [82].

Family planning services/safe contraception and preconception counseling: for women of reproductive age, coordinated care is essential to address contraception choices, preconception counseling, and pregnancy management. Many psoriasis medications are contraindicated during pregnancy, so advance planning with a healthcare provider can optimize reproductive outcomes and minimize disease activity during pregnancy [59,65].

## 7. Oral Contraceptives and Psoriasis

Oral contraceptives (OCs) can influence psoriasis due to their impact on hormonal levels, particularly estrogen and progesterone. Hormones play a significant role in modulating immune responses, which are central to the development and progression of psoriasis [9,10]. In some cases, oral contraceptives may have positive effects on psoriasis by stabilizing hormonal fluctuations [6]. Psoriasis symptoms in women are often influenced by changes in hormone levels during menstruation, pregnancy, or menopause [30,33,38]. Estrogen-containing OCs can help stabilize these fluctuations, potentially reducing the severity of symptoms. Estrogen may also exert an anti-inflammatory effect by modulating cytokines and reducing the activity of pro-inflammatory immune cells, such as Th17 and Th1 cells [16]. Additionally, estrogen has been shown to enhance skin barrier function and hydration, which may alleviate certain symptoms of psoriasis, particularly in postmenopausal women or those with dry skin [83].

However, oral contraceptives can also have negative effects on psoriasis in some individuals. Progestins, the synthetic progesterone component of OCs, may have a pro-inflammatory effect, potentially aggravating psoriasis symptoms [19,20,21,84]. The specific type of progestin used in a contraceptive can influence its impact, with newer-generation progestins possibly being less inflammatory than older formulations. Furthermore, not all women respond to oral contraceptives in the same way, and some may experience worsening symptoms due to genetic or epigenetic factors affecting their hormonal response. Discontinuing OCs can also lead to a rebound effect, with hormonal fluctuations potentially triggering psoriasis flare-ups [84,85].

When considering oral contraceptives for women with psoriasis, several factors need to be taken into account. Low-estrogen formulations or those with newer-generation progestins may be better tolerated. Additionally, women with severe psoriasis or psoriatic arthritis may be advised to use non-hormonal methods of contraception to avoid potential exacerbation of symptoms [84,85,86]. For women taking teratogenic treatments, such as methotrexate or acitretin, effective contraception is critical to prevent pregnancy during treatment, and OCs are often recommended under careful medical supervision [59].

It is important to recognize that women with psoriasis are at higher risk for metabolic syndrome and cardiovascular disease, which should be considered when prescribing estrogen-containing contraceptives due to their potential to increase the risk of thromboembolism [59,64]. Women considering oral contraceptives should consult both a dermatologist and a gynecologist to determine the safest and most effective option for their individual needs. Close monitoring for changes in psoriasis symptoms after starting or stopping OCs is essential to address any potential complications promptly. Combining hormonal stabilization with lifestyle modifications, such as stress management and an anti-inflammatory diet, may further optimize psoriasis outcomes [59,64,71,83,86].

Although oral contraceptives can play a role in managing psoriasis for some women, there are still significant research gaps. Rigorous clinical trials are needed to evaluate the long-term effects of different types of OCs on psoriasis severity, and more studies are required to understand the molecular mechanisms by which estrogen and progestin influence the disease. Personalized approaches to contraceptive and psoriasis management, including the identification of biomarkers that predict an individual’s response to OCs, may ultimately improve outcomes for women with psoriasis.

## 8. Future Directions and Research

Recent research highlights the complexity of psoriasis, a chronic inflammatory disease that appears to be influenced by various factors, including immune function, hormonal balance, genetics, and psychological aspects [1,5,6,75]. Below are key areas for further investigation.

### 8.1. Estrogen Receptor Studies

Role of estrogen receptors in psoriasis pathogenesis: estrogen has been shown to influence immune function, but the exact mechanisms remain unclear [6,10]. Estrogen receptors (ERs), specifically ERα and ERβ, are expressed on immune cells and play roles in immune regulation. Research suggests that ERα activation might have pro-inflammatory effects, while ERβ activation could be anti-inflammatory, potentially impacting psoriasis severity [11,87].

Targeting estrogen receptor pathways: by investigating how different estrogen receptors influence immune cells, scientists may uncover specific pathways that could be modulated for therapeutic purposes. For example, selective estrogen receptor modulators (SERMs) might offer ways to manage inflammation in psoriasis without the side effects associated with broad hormone replacement therapies [88].

### 8.2. Genetic and Epigenetic Factors

Genetic variations and psoriasis susceptibility: studies have identified several genetic loci associated with psoriasis, particularly in genes related to immune responses, such as IL-23 and TNF-α pathways [89]. Research into how these genetic variations interact with hormonal changes could explain why psoriasis severity fluctuates with life stages (e.g., puberty, pregnancy, menopause) [7,9,19,89].

Epigenetic modifications and hormone sensitivity: epigenetic changes, such as DNA methylation and histone modifications, influence gene expression without altering DNA sequences [89]. Hormones like estrogen can impact these epigenetic mechanisms, potentially affecting psoriasis severity and treatment responses [16,90,91]. Investigating how epigenetic factors modify hormonal sensitivity in individuals with psoriasis may provide insights into why some patients experience more severe symptoms and aid in the development of personalized therapies.

Personalized medicine: identifying genetic and epigenetic markers linked to psoriasis severity and treatment response could facilitate personalized medicine [91]. By tailoring treatment approaches based on genetic profiles, healthcare providers may be able to optimize therapies and reduce side effects.

### 8.3. Longitudinal Studies

Natural history of psoriasis through life stages: longitudinal studies that follow women through hormonal transitions (e.g., puberty, pregnancy, menopause) are essential for understanding how hormonal fluctuations impact psoriasis [7]. Such studies can clarify if specific life stages exacerbate or alleviate symptoms, informing the timing and types of intervention strategies.

Tracking disease progression and triggers: by monitoring psoriasis progression and flare-up patterns in relation to hormonal changes, researchers can gather insights into which interventions are most effective at different life stages. For instance, such studies could identify whether hormone fluctuations are linked to periods of remission or exacerbation, guiding both timing and choice of treatments.

Evidence-based treatment timelines: long-term data can help identify optimal treatment windows and strategies for managing psoriasis, potentially leading to interventions that are timed to life-stage transitions, such as beginning hormone-based treatments at menopause if appropriate.

### 8.4. Hormone-Based Treatments

Selective estrogen receptor modulators (SERMs): SERMs, which are already used in conditions like osteoporosis and breast cancer, may represent a promising area for psoriasis treatment [81,92]. These drugs selectively stimulate or block estrogen receptors, which could allow for modulation of immune responses related to psoriasis without widespread hormonal effects [81]. Trials are needed to determine the efficacy and safety of SERMs for psoriasis.

Progesterone analog research: progesterone, another key hormone, may have immunomodulatory effects that could impact psoriasis [9]. Progesterone analogs could be explored for their potential in modulating immune function, particularly during life stages where progesterone naturally fluctuates (e.g., pregnancy). Current research is limited, but understanding how progesterone and its receptors impact psoriasis could open new therapeutic options.

Clinical trials for hormone-based therapies: clinical trials investigating hormone-based therapies, including estrogen and progesterone treatments, are essential for understanding their effects on psoriasis [6,9]. Such trials can assess the safety, efficacy, and optimal dosing regimens of hormone therapies for patients with psoriasis, especially for women undergoing hormonal changes.

### 8.5. Psychoneuroimmunology Research

Mind–body interactions in psoriasis: the emerging field of psychoneuroimmunology (PNI) examines how psychological factors, neurological processes, and immune responses interact in disease contexts [93]. Stress, for example, can exacerbate psoriasis by stimulating the hypothalamic–pituitary–adrenal (HPA) axis, which releases cortisol and other stress hormones. Chronic stress also impacts immune function, leading to increased inflammatory cytokine production that can worsen psoriasis [94].

Role of stress hormones and neurotransmitters: research suggests that stress hormones like cortisol, as well as neurotransmitters such as serotonin and dopamine, may influence immune responses in psoriasis [26,27]. This connection indicates that stress reduction and psychological interventions may play important roles in managing psoriasis, especially given the skin–brain connection [95]. Investigating these pathways could lead to novel, holistic approaches that combine pharmacological and psychological therapies.

Interdisciplinary treatment approaches: given the complex interactions between the mind and immune function, interdisciplinary approaches that include mental health support alongside dermatologic treatment may improve outcomes. This approach might involve cognitive-behavioral therapy (CBT), mindfulness-based stress reduction, and other interventions aimed at reducing stress and improving mental well-being [73,74,82].

## 9. Conclusions

Hormonal balance significantly influences the course and severity of psoriasis in women, particularly during key life stages such as the menstrual cycle, pregnancy, postpartum period, and menopause [7,8]. Estrogen and progesterone play pivotal roles in modulating immune responses central to psoriasis pathogenesis, with fluctuations in their levels often correlating with symptom exacerbations or improvements [6,9]. Understanding these hormonal effects is essential for optimizing management strategies.

The study underscores the importance of a personalized, multidisciplinary approach to managing psoriasis in women. By integrating medical therapies—including safe use of biologics, topical treatments, and hormone-based interventions—with lifestyle modifications, psychological support, and patient education, healthcare providers can address the physical, emotional, and social impacts of the disease. Tailoring treatment strategies to align with hormonal changes can significantly improve outcomes and empower women to navigate the complexities of living with a chronic autoimmune condition. Ongoing research into hormonal mechanisms, receptor-specific therapies, and psychoneuroimmunological interactions is vital for advancing targeted treatments. Longitudinal studies tracking hormonal influences across life stages will further inform evidence-based clinical practices, ultimately enhancing the quality of life for women with psoriasis. These efforts reflect the necessity of aligning scientific exploration with individualized patient care.

## Data Availability

No new data were created or analyzed in this study. Data sharing is not applicable to this article.

## Abbreviations

TNF-α	Tumor Necrosis Factor-alpha.
IL	Interleukin.
IFN-γ	Interferon-gamma.
TGF-β	Transforming Growth Factor-beta.
Th	T-helper cells.
Tregs	Regulatory T cells.
HPA axis	Hypothalamic–Pituitary–Adrenal axis.
HPO axis	Hypothalamic–Pituitary–Ovarian axis.
PASI	Psoriasis Area Severity Index.
PCOS	Polycystic Ovary Syndrome.
OCs	Oral Contraceptives.
HRT	Hormone Replacement Therapy.
PUVA	Psoralen plus ultraviolet A therapy.
UVB	Ultraviolet B therapy.
ER	Estrogen Receptor.
SERMs	Selective Estrogen Receptor Modulators.
CBT	Cognitive-Behavioral Therapy.
DNA	Deoxyribonucleic Acid.
FDA	Food and Drug Administration.
